# Technical note: A PET/MR coil with an integrated, orbiting 511 keV transmission source for PET/MR imaging validated in an animal study

**DOI:** 10.1002/mp.15586

**Published:** 2022-03-08

**Authors:** Andreas Renner, Ivo Rausch, Jacobo Cal Gonzalez, Elmar Laistler, Ewald Moser, Thies Jochimsen, Tatjana Sattler, Osama Sabri, Thomas Beyer, Michael Figl, Wolfgang Birkfellner, Bernhard Sattler

**Affiliations:** ^1^ Center for Medical Physics and Biomedical Engineering Medical University Vienna Vienna Austria; ^2^ Department of Radiation Oncology Medical University Vienna Vienna Austria; ^3^ Department of Nuclear Medicine University Hospital Leipzig Leipzig Germany; ^4^ Clinic for Ruminants and Swine University of Leipzig Leipzig Germany

**Keywords:** attenuation correction, MRI, PET, post‐injection, transmission source

## Abstract

**Background:**

MR‐based methods for attenuation correction (AC) in PET/MRI either neglect attenuation of bone, or use MR‐signal derived information about bone, which leads to a bias in quantification of tracer uptake in PET. In a previous study, we presented a PET/MRI specific MR coil with an integrated transmission source (TX) system allowing for direct measurement of attenuation. In phantom measurements, this system successfully reproduced the linear attenuation coefficient of water.

**Purpose:**

The purpose of this study is to validate the TX system in a clinical setting using animals and to show its applicability compared to standard clinical methods.

**Methods:**

As test subject, a 15‐kg piglet was injected with 53 MBq of 18F‐NaF. The μ‐map obtained with the TX system and the reconstructed activity distribution were compared to four established AC methods: a Dixon sequence, an ultra‐short echo time (UTE) sequence, a CT scan, and a 511 keV transmission scan using a Siemens ECAT EXACT HR+ as the reference. The PET/MRI measurements were performed on a Siemens Biograph mMR to obtain the μ‐map using the TX system as well as the Dixon and UTE sequence directly followed by the CT and ECAT measurements.

**Results:**

The reconstructed activity distribution using the TX system for AC showed similar results compared to the reference (<5% difference in hot regions) and outperformed the MR‐based methods as implemented in the PET/MRI system (<10% difference in hot regions). However, the additional hardware of the TX system adds complexity to the acquisition process.

**Conclusion:**

Our porcine study demonstrates the feasibility of post‐injection transmission scans using the developed TX system in a clinical setting. This makes it a useful tool for PET/MRI in cases where transmission information is needed for AC. Potential applications are studies using larger animals where state‐of‐the‐art atlas‐based or artificial intelligence AC methods are not available.

## 1 INTRODUCTION

Attenuation correction (AC) is a standard procedure to ensure accurate quantification of tracer uptake in positron emission tomography (PET). Depending on the body size, AC is also significant in small animal PET.^1^, ^2^ The impact of photon interactions on the reconstructed images is substantially less in mice or rats than in the human body.^2^ Still, without AC, the reconstructed activity concentration is underestimated by 10%–20% in mice and 20%–40% in rats.^1^, ^3^ For comparison, in an adult human, this can be as high as 80%–99%.^4^

In animals with a diameter smaller than 4 cm,^2^ assuming a constant attenuation coefficient (e.g., water) within the body contour is sufficient. This method is called uniform AC.^3^ For larger animals, additional information is needed. This can be obtained using transmission information of either integrated transmission sources (e.g., Cs‐137 source in the commercial standalone PET system ClairvivoPET^5^, ^6^), Ge‐68 line sources (clinical standalone PET system ECAT HR+), or an additional CT image (hybrid PET/CT systems).

In a combined PET/MRI system, transmission information is not available, and AC has to be based on the MR signal. MR‐based AC (MRAC) methods alone typically neglect bony structures leading to an underestimation of tracer concentration, particularly in the head.^7^, ^8^, ^9^, ^10^ MR‐invisible objects such as MR coil materials and the patient bed add to this effect.^11^

In humans high quality MRAC can be achieved using MR sequences with short or zero echo time—for example, ultra‐short echo time (UTE)—as well as atlas‐based and deep learning supported methods.^12^, ^13^, ^14^, ^15^, ^16^, ^17^ An extensive overview of AC and artificial intelligence (AI)‐based AC is given in reference [18]. AI‐based AC methods utilize MR data^19^, ^20^ as well as the PET emission data.^21^ All these MRAC‐ and AI‐based methods are tailored to the application in humans or even specific body regions, for example, the brain. Translation to other body regions and especially to animals is not straight forward and can lead to artifacts in the map of the attenuation coefficients μ (μ‐map) and, thus, in the reconstructed activity distribution.

A possible solution is the direct measurement of attenuation using a transmission scan. Several groups have already reported using static transmission sources.^22^, ^23^ In this study, we use an orbiting transmission source (TX) system integrated in an MR coil for PET/MRI to perform accurate AC in non‐time‐of‐flight (ToF) PET. The system is described in detail in references [24, 25]. The TX system was previously validated in a phantom study.^24^ To obtain validation of the TX system in a clinical setting, an animal study was conducted in the Department of Nuclear Medicine at the University Hospital Leipzig in Germany. The μ‐map obtained with the TX system was compared to standard MRAC methods (Dixon and UTE) and to the μ‐map obtained from a separately acquired CT. As a reference, a transmission scan using 511 keV annihilation photons from ^68^Ga/^68^Ge line sources in a stand‐alone PET system was used. The transmission scan was used as reference as it provides a direct measurement of the attenuation.

## 2 MATERIALS AND METHODS

The animal experiment was approved by the responsible institutional and federal state authorities (Landesdirektion Leipzig; TVV 08/13): the piglet (age: ∼6 weeks, weight: 15 kg) fasted on the day of imaging and received intramuscular injection of 1 ml azaperone and 4 ml ketamine to introduce anesthesia. After 15 min, 2 ml of ketamine and 1 ml of midazolam (5 mg/ml) was injected intravenously (ear vein, V. auricularis), followed by 5 ml G40, 3 ml ketamine, and 1.5 ml midazolam in 50 ml NaCl 0.9% with an infusion pump at a flow rate of 37.5 ml/h to maintain the narcosis throughout the whole investigation time. During narcosis, the animal maintained spontaneous respiration and no mandatory ventilation was applied.

For PET imaging, the animal was injected with 53 MBq of ^18^F‐labeled sodium fluoride (NaF). The activity values given were determined using a cross‐calibrated dose calibrator and are decay corrected to mid acquisition duration.

### 2.1 Measurement procedure

The piglet was measured in three different imaging systems (in given order), namely, a:

- Siemens Biograph mMR PET/MRI system using the TX system to obtain emission and transmission PET data as well as MRI data of a Dixon and a UTE sequence for MRAC;
- Siemens ECAT EXACT HR+ stand‐alone PET system to obtain a 511 keV transmission scan;
- Siemens Biograph 16 CT system to obtain a scan for CT‐based AC using bilinear scaling.^26^

The piglet was positioned in prone position with the legs alongside the body on a custom‐made plastic trough including a piglet head‐holder to facilitate transportation between different imaging sites and modalities. Rigid foamed plastic rest, form‐fitted to the piglet's head, was used to ensure consistent positioning within the MR coil in the PET/MRI.

#### 2.1.1 TX system transmission scan

As transmission source, a refillable container was filled with liquid solution containing ^18^F as positron emitter. Prior to the PET acquisition of the piglet, a 30 min blank scan (i.e., a scan without any object in the TX system) with a transmission source activity of 175 MBq was acquired.

A 30 min emission scan of the piglet was acquired in the PET/MRI with the transmission measurement performed simultaneously. The total transmission scan time was 12 min using a transmission source activity of 125 MBq. The transmission scanning consisted of 12 individual transmission scans with a duration of 1 min each. Between each individual transmission scan, there was a 1 min break to acquire emission‐only data as well as to reverse the flow direction of the hydraulic system. The ratio of the blank and transmission scan was reconstructed using an ordered subset expectation maximization (OSEM) algorithm. The emission‐only data as well as sinogram windowing were used to separate emission from transmission data and to reduce both scatter and random coincidences in the transmission data. More details about the reconstruction of the μ‐map are given in reference [24]. As the transmission scan is recorded with the PET detector itself, the μ‐map inherently has the same resolution as the PET emission image. The μ‐map is reconstructed with a slice thickness of 2 mm and an in‐plane resolution of 2.1 mm × 2.1 mm.

#### 2.1.2 ECAT transmission scan

The Siemens ECAT EXACT HR+ PET system has an axial field of view (FoV) of 15.5 cm. As this is shorter than the axial FoV of the Siemens Biograph mMR PET/MRI system of 25.8 cm, a transmission scan with two bed positions was acquired. The scan time per bed position was 10 min with a total transmission source activity of 81 MBq. The transmission system of this PET device consists of three ^68^Ga/^68^Ge rod sources. The μ‐map was reconstructed using an OSEM algorithm as implemented by the vendor. The reconstructed μ‐map has an isotropic resolution of 5.15 mm.

### 2.2 Reconstruction of activity distribution

The e7‐tools for mMR from Siemens were used for the reconstruction of the activity distributions. The μ‐maps obtained from CT and ECAT were co‐registered to the UTE μ‐map using Amide^1^. The μ‐maps based on Dixon and UTE sequences as well as the μ‐map obtained with our TX system are inherently co‐registered and, therefore, share the same orientation and coordinates as the emission data of the PET/MRI acquisition and did not require co‐registration. The μ‐maps obtained from the CT and the ECAT needed co‐registration before they could be applied for AC. As target for this registration, the μ‐map acquired with the UTE‐sequence was used because of the similar image content compared to CT and ECAT.

A μ‐map of the TX hardware itself was obtained using a high‐resolution CT and bilinear scaling.^26^ After registration to the non‐AC emission PET image, it was added to all μ‐maps to account for the photon attenuation of the added hardware in the PET FoV.

### 2.3 Data analysis

To evaluate the performance of our proposed AC method, the reconstructed μ‐maps as well as the reconstructed activity distributions obtained with all five AC methods were compared.

The reconstructed μ‐maps were visualized using the native spatial resolution of each imaging modality to illustrate the main differences.

**FIGURE 1:**
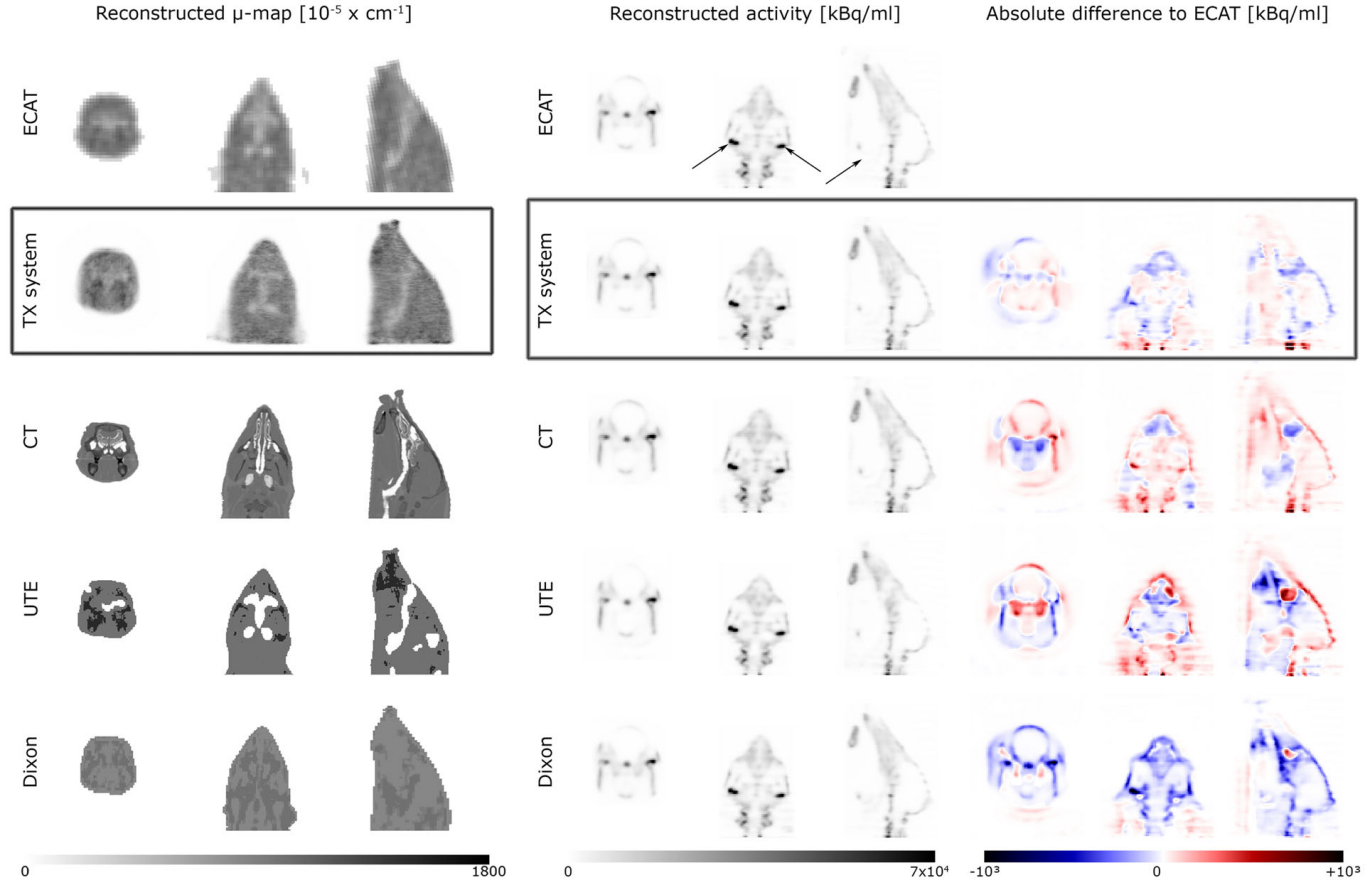
Reconstructed μ‐maps (left) and activity distributions (middle and right): Rows from top to bottom show transversal/coronal/sagittal planes of all five AC methods (transmission scan using the ECAT system, TX system, CT‐based AC, UTE and Dixon) used; results using the TX system are highlighted by a black frame. The grey‐value of the reconstructed μ‐maps corresponds to the LAC in [10^–5^ cm^–1^]; images are given in the original resolution of each modality. In the reconstructed activity distribution (middle) of the 18F‐NaF PET scan of the piglet, the locations of the ROIs used for the quantitative evaluation are indicated in the ECAT image (both ROI_hot_ in the coronal plane and the ROI_cold_ in the sagittal plane). The right side shows the difference to the reference (ECAT). The horizontal stripes in the neck area originate from a slight shift of the hose of the liquid drive caused by transportation of the TX system from Vienna to Leipzig (the high resolution CT of the TX system for AC correction of the hardware was acquired in Vienna)

The reconstructed activity distributions were evaluated in three regions of interest (ROIs) using ECAT as reference (ROI positions are indicated in Figure 1):

- ROI_hot, left_ and ROI_hot, right_ with a size of 27 voxels each were placed in the two regions with the highest tracer concentration;
- ROI_cold_ with a size of 198 voxels was placed in the trachea, where no tracer concentration is expected.

For the evaluation, the mean and standard deviation of the reconstructed activity concentration of the respective ROIs and the relative difference of the mean activity concentration to the reference (ECAT) were used.

In addition, the difference to the reference (ECAT) was calculated for visual inspection for each AC method. Instead of relative difference, the difference was chosen here as a metric based on the characteristics of the tracer used. 18F‐NaF typically shows uptake throughout the skeleton. Using relative difference for visualization would, thus, particularly highlight areas with low tracer uptake.

## 3 RESULTS

### 3.1 Reconstructed μ‐maps

The reconstructed μ‐maps (Figure 1, left) show the superior spatial resolution of CT compared to all other methods. Bones and respiratory tracts are distinct. The same is true for the UTE‐sequence, but in this case, there is obvious mis‐segmentation between tissue types (bone, air, soft tissue) at several locations (the maxillary bone is frayed, the size of the trachea augmented) and in the region of the eye‐bulb, tissue is mis‐segmented as air. The Dixon sequence replaces bone and air inside the body by tissue. Both 511 keV transmission images (from ECAT and TX system) have low spatial resolution compared to CT, but still, the trachea and major bone structures can be visually identified.

The reference for the body boundaries is given by the CT. In the μ‐maps obtained from the ECAT and the TX system, the size of the head is successfully reproduced, but the edges are blurred due to scatter and the lower spatial resolution. Both MR sequences showed mis‐segmentation of tissue as air near the body boundaries. This was especially the case for the Dixon sequence at the trunk of the pig.

### 3.2 Reconstructed activity distributions

The reconstructed activity concentration (Figure 1, middle) for the reference of the left and right hot ROI was 73 kBq/ml ± 25 kBq/ml and 70 kBq/ml ± 26 kBq, respectively (compare Table 1 for all values). The ROI in the trachea showed an activity concentration of 1 kBq/ml ± 1 kBq

**TABLE 1:** Evaluation of reconstructed activity distribution

ROI	Modality	Activity [kBq/ml]	Rel. difference
ROI_hot left_	ECAT	73 ± 25	−
	CT	82 ± 28	+12%
	TX system	73 ± 25	0%
	Dixon	68 ± 23	−7%
	UTE	75 ± 25	+3%
ROI_hot right_	ECAT	70 ± 26	−
	CT	81 ± 29	+16%
	TX system	73 ± 27	+4%
	Dixon	66 ± 24	−6%
	UTE	73 ± 27	+4%
ROI_cold_	ECAT	1.1 ± 0.8	−
	CT	1.3 ± 1.2	+18%
	TX system	1.1 ± 0.8	0%
	Dixon	2.1 ± 1.5	+91%
	UTE	0.4 ± 0.4	−64%

*Note*: The activity concentration is given for three ROIs; two in high uptake regions (ROI_hot_) and one in the trachea (ROI_cold_). The location of the ROIs is indicated in the top middle part of Figure 1.

The result using the Dixon sequence predominantly shows an underestimation of tracer uptake (−7% and −6% for left and right hot ROI), except for the ROI in the trachea and a region around the eye‐bulb (compare right side of Figure 1). The result using the UTE‐sequence shows both considerable over‐ and underestimation of tracer uptake. Using CT‐based AC, the result most notably showed overestimation of tracer uptake, especially near bony structures. As 18F‐NaF shows high uptake in the skeleton, results of the CT‐based AC showed the biggest difference in the hot ROIs (+12% and +16%). However, the differences are generally smaller than using the MRAC methods. The reconstructed activity distribution using the TX system for AC showed the least difference to the reference.

## 4 DISCUSSION

The animal study demonstrated the applicability of the novel TX system in a (pre‐)clinical setting. To the authors knowledge, it is the first successful non‐ToF PET/MRI transmission scan in a living subject. The proposed transmission‐based AC method yielded a more realistic μ‐map than the MRAC methods currently used in clinical routine. Also, the reconstructed activity distribution showed the least difference to the reference as the proposed method constitutes a direct measurement of attenuation.

CT allows for much higher spatial resolution as compared to PET images. Thus, the CT μ‐maps need down‐sampling to match the lower resolution before they can be applied to the PET data for AC. Hence, the lower resolution of the μ‐map of our TX system compared to CT is not a disadvantage for AC.

The added activity from the transmission source does not compromise the count rate performance of the PET/MRI system for measurements with an emission activity well below the maximum NECR of the system.^24^ However, a measurement with an emission activity close to the NECR would require a pre‐injection TX scan leading to reduced patient throughput.

The μ‐map obtained from the Dixon sequence yielded an underestimation of tracer concentration in the reconstructed activity distribution. This is due to the absence of bone in the μ‐map. The μ‐map obtained from the UTE‐sequence yielded a considerable over‐ and underestimation of the tracer uptake depending on the region. This can be explained by mis‐segmentation of different tissue types intrinsic to this method.^27^, ^28^, ^29^ In the case of the CT‐based AC, a minor overestimation of activity concentration in regions with high attenuating tissue (e.g., cortical bone) was observed. This is a known aspect of converting CT data to the LAC at 511 keV using bilinear scaling.^30^, ^31^ This bias is still present when using tri‐linear scaling.^32^ Especially for longitudinal studies, this effect has to be considered to avoid interchangeable use of CT‐based and TX‐based AC, which could introduce spurious differences in the reconstructed activity concentration.

Post‐injection transmission scanning allowed for an acquisition of the μ‐map simultaneous with the PET emission measurement. Thus, the PET/MRI acquisition time did not exceed a standard (clinical) acquisition. However, the increased complexity of the acquisition process due to additional hardware and more complex preparations needed may be a disadvantage if translation into clinical routine is intended. Especially, shifts of the hose of the liquid drive have to be avoided to prevent artifacts in the reconstructed μ‐maps.

Potential applications of the prototype TX system are PET/MRI studies using larger animals (e.g., pig or sheep), as in these cases, attenuation is not negligible and state‐of‐the‐art atlas‐based AC methods are not applicable. AI‐based AC methods would require a substantial amount of training data which are laborious to gather for a variety of animals. Typically, an additional CT is acquired for AC as MRAC is particularly error‐prone in animal studies. However, this additional acquisition requires transportation between the imaging systems and introduces the risk of mis‐positioning of the animal and, as a result, registration errors between CT and PET data. These limitations can be overcome with the proposed TX system.

The next step of validation of the novel TX system should be post‐injection measurements in humans. In that case, atlas‐based methods would be available, and a comparison of the proposed AC method would be possible.

## 5 CONCLUSION

The porcine study demonstrates the feasibility of post‐injection transmission scans in a (pre‐) clinical setting. The novel TX system performed comparable to an established transmission source method of a clinical standalone PET system.

## CONFLICT OF INTEREST

The authors declare no conflict of interest.

## Footnotes

1 Amide is a free software tool for medical image analysis (http://amide.sourceforge.net/).
